# Xerogels Morphology Details by Multifractal Analysis and Scanning Electron Microscopy Images Evaluations of 5-Fluorouracil Release from Chitosan-Based Matrix

**DOI:** 10.3390/gels8120820

**Published:** 2022-12-12

**Authors:** Maria-Alexandra Paun, Mihai-Virgil Nichita, Vladimir-Alexandru Paun, Viorel-Puiu Paun

**Affiliations:** 1 School of Engineering, Swiss Federal Institute of Technology (EPFL), 1015 Lausanne, Vaud, Switzerland; 2 Division Radio Monitoring and Equipment, Section Market Access and Conformity, Federal Office of Communications (OFCOM), 2501 Bienne, Canton of Bern, Switzerland; 3 Doctoral School, Faculty of Applied Sciences, University Politehnica of Bucharest, 060042 Bucharest, Romania; 4 Five Rescue Research Laboratory, 75004 Paris, France; 5 Physics Department, Faculty of Applied Sciences, University Politehnica of Bucharest, 060042 Bucharest, Romania; 6 Academy of Romanian Scientists, 50085 Bucharest, Romania

**Keywords:** 5-fluorouracil, chitosan hydrogels, multifractal dynamics, SEM images, fractal analysis

## Abstract

Four medicament delivery formulations based on 5-fluorouracil in a chitosan substantial matrix were realized in situ via 3,7-dimethyl-2,6-octadienal element hydrogelation. Representative samples of the final realized compounds were investigated from an analytic, constitutional, and morphological viewpoint via Fourier transform infrared (FTIR) spectroscopy and scanning electron microscopy (SEM). The SEM images of the formulations were investigated in concordance with fractal analysis, and the fractal dimensions and lacunarity were computed. The developed mathematical multifractal model is necessarily confirmed by the experimental measurements corresponding to the 5-fluorouracil release outside the chitosan-formed matrix.

## 1. Introduction

Polymer networks with hydrophilic characteristics are called hydrogels. These hydrogels are a type of soft, wet material with a three-dimensional network of crosslinked polymers that hold water in intermolecular spaces. Polymers are commonly applied in the design and manufacturing of hydrogels. The materials used for the development of hydrogels are roughly classified into two types based on their origin: natural and synthetic polymers. Dried gel beads in the form of xerogels, cryogels, or aerogels are prepared using different gelation conditions (aqueous and ethanolic CaCl_2_ solutions) and drying methods (supercritical drying, freeze drying, and oven drying) to obtain particles with a broad range of physicochemical and textural properties. Xerogels and cryogels are obtained after the atmospheric drying and freeze drying of gels, respectively.

Xerogel is a solid gel obtained by drying with unhindered shrinkage. The drying process of xerogels involves solvent evaporation under standard conditions using conventional methods. Xerogels are mesoporous materials with high thermal stability. Significantly, xerogels are non-toxic, cost-effective, and biocompatible; they also have a large surface area and high porosity, and can easily be modified.

Cryogels, which are materials with a macroporous three-dimensional structure, are produced as a result of controlled freezing during polymerization with a highly interconnected polymer network. Cryogels are used for materials that extract liquid at a much lower temperature using a vacuum (sublimation phase of the solvent).

Aerogel can be defined as a solid that forms from a gel by drying at a supercritical state. The drying process in aerogels can be described as freeze drying, supercritical drying, or ambient pressure drying. Aerogel is a lightweight material and exhibits a large surface area that ranges between 200 and 1000 m^2^/g. It has many important properties such as a large amount of controlled pore size distribution, high conductivity, transparency, low density, flexibility, low dielectric constant, and high mechanical strength.

Aerogel and xerogel are important types of solid gel materials. The key difference between aerogel and xerogel is that aerogel forms when the liquid from the gel is extracted at a supercritical state, whereas xerogel forms when the liquid from the gel is evaporated at room temperature. Moreover, aerogel has a comparatively larger surface area than xerogel. Cryogel is a commercialized name for a product developed by Aspen Aerogels. It is a flexible aerogel composite blanket, designed for insulating cold temperature environments ranging from cryogenic to ambient.

Xerogels have a solid consistency, and usually possess properties such as a greater porosity and larger surface area in conjunction with reduced pore dimensions [1]. Regarding the substances presented above, in this article, we will prioritize chitosan. Chitosan is a significant biopolymer; it is plentiful in nature, cationic (positively charged), and non-immunocompetent, and has reduced toxicity and unlimited organic sustainability [2]. The comparative apparition of the two monomeric construction elements (D-glucosamine and N-acetyl-glucosamine) is decisive to whether chitosan is mostly an ampholyte or a polyelectrolyte dominated by acidic pH values. The chemical structure is not only critical for the activity conditions localized at the surface, but is essential for sol–gel transition, which occurs frequently. In addition, chitosan, based on its stable character, is frequently utilized in industry, as well as in agriculture, food, and cosmetics [3,4]. This chemical compound has excellent moisture capture, moisture restraint, opsonization, and bacterial inhibition. It is applicable to different cosmetic products, such as moisturizing cream, shower gel, cleaning cream, mousse, and advanced ointment frost. In addition, it is applicable as an emollient and antistaling constitutive element for alimentation, vegetables, and fruits, and as a flocculant for wastewater treatment due to its medicament continuous release factor and innocuous toxicity. It is also frequently used as a dyeing–printing assistant in paper fabrication [5,6,7,8,9].

The second chemical element used in this study is called 5-fluorouracil. Chemically speaking, 5-fluorouracil is an element analog with uracil, and has a fluorine atom in the C-5 location [8]. As a stand-alone medicine, it can be administered both intravenously and orally. In the case of intravenous route administration, the 5-FU drug may be quickly inserted in the target cell, utilizing an identical transport mechanism to that of uracil. Administered orally, in the form of pro-medicaments (oral FPs), 5-fluorouracil is defective and disturbs bioavailability due to DPYD function variability. Accordingly, this behavior is appropriate for unpredictable levels of 5-fluorouracil in the blood plasma of patients [9].

5-fluorouracil-encapsulated chitosan nanoparticles were realized for the investigation of spatially delimited medicament release as a result of pH chitosan nanoparticles’ sensibility. The chitosan-covered magnetic nanoparticles were utilized to expand the delivery of 5-fluorouracil. Chitosan is a cationic polymer. Chitosan nanoparticle size was found to be below 100 nm by Zetasizer, transmission electron microscopy (TEM), and field scanning electron microscopy (FSEM) results.

An accumulation of recent evidence has proven that the 5-fluorouracil transportation process could be passively triggered by paracellular and transcellular monolayer tumoral cells. As a demonstrated alternative, 5-fluorouracil could probably cross/exceed the so-called blood–brain obstacle via passive diffusion. Furthermore, 5-fluorouracil can convert moving metabolites into target items.

Hydrogels are important medicament release systems that increase bioavailability and can be used as implantable medicament storage systems, permitting large medicament concentrations to be provided bluntly to suffering tissue, defeating the weak bioavailability problem and diminishing the proposed goal effects. In the current situation, hydrogels afford countless benefits over solid medicament storage implants because they possess physicochemical characteristics alike to natural tissues of living matter, mitigating rash reactions in circumambient tissue after implantation [10,11].

In accordance with tissue engineering expectations and recommendations, a multi-compatibility chitosan is an interesting option for matrix polymers [12,13]. Thus, chitosan hydrogels are sought after for the controlled restricted release of medicaments, which makes them extremely useful for our study [14].

At the moment, it is assumed that chitosan nanoparticles could avert the therapeutic side effects induced by 5-fluorouracil at administration and later in the process. Thereby, 5-FU-uploaded chitosan nanoparticles may be utilized as efficient medicament release systems, and chitosan–medicament pairs are proposed to defeat the complicated side effects caused by 5-FU presence. However, the pH sensibility of chitosan nanoparticles regarding 5-fluorouracil delivery and the subsequent mathematical modeling of deliverance kinetics has not yet been fully experimentally confirmed [15,16].

In this study, considering compartmental logical circumstances, a new procedure is advanced, taking into consideration medicament delivery dynamics in complicated real systems—recognized as effective in pharmacokinetics studies—considering that drug release dynamics can be depicted by multifractal curves [17,18]. Presuming that the implicated configurational unit dynamics of polymer–medicament pairs occur on the multifractal curves (which are continuous and nondifferentiable), this shows that in 1D hydrodynamic conventional multifractal variables, the medicament delivery process (known as Fickian diffusion or non-Fickian diffusion) [17] is produced via isochronous dynamics, depending on the change from differentiable curves to non-differentiable curves [19].

Through the experimental measurements carried out, the model has been certified. In addition, we must highlight the application of fractal analysis to the interpretation of the morphology of the samples investigated by SEM imaging. Thus, the fractal dimensions and the lacunarity of the SEM images were calculated and introduced as a voxel configuration, expressing the values of a normal network in a 3D space. These results are presented to confirm the fractal behavior of the studied drug release process.

## 2. Results and Discussion

### 2.1. Theoretical Part: Delivery Kinetics Mathematical Modeling

The polymer–medicament theoretical model of compound deliverance dynamics can be depicted by continuous curves with non-differentiable functions (named multifractal curvatures) [19,20]. Thus, the multifractal motion theory presented here in hydrodynamic configuration reaches functionality via the following equations [21,22]:

(1) $$\frac{\partial }{\partial t}V_{D}^{i}+V^{l}\frac{\partial }{\partial X^{l}}V_{D}^{i}=-\frac{\partial }{\partial X^{i}}Q$$

(2) $$\frac{\partial }{\partial t}\rho +\frac{\partial }{\partial X^{l}}\left(\rho V_{D}^{l}\right)=0$$

where i,l=1,2,3.

In Equation (1)

(3) $$Q=\lambda^{2}{\left(dt\right)}^{\left[\frac{2}{f\left(\alpha \right)}\right]-1}\frac{1}{\sqrt{\rho }}\frac{\partial }{\partial X^{l}}\left(\frac{\partial }{\partial X^{l}}\right)\sqrt{\rho }$$

In Equations (1)–(3), the nonfractal (classic) time *t* has an affine parameter role of the append deliverance function, *X^l^* is one of the multifractal 3D coordinates, and $V_{D}^{l}$ is the “multifractal fluid” speed of differentiable scale resolution (the polymer–medicament binary system is assumed to be a “multifractal flowing substance”) [23,24]. In continuation, ρ is the “multifractal fluid” state density, λ is the configurational constant associated with the deliverance procedure related to the fractal–multifractal specific passage, *dt* is the scale resolution factor and *f*(*α*) is the α order singular spectrum contingent based on the calculated fractal dimension.

The differential Equations (1) and (2) permit, in the mathematical 1D situation—together with a set of distinctly defined boundary conditions and initial conditions [25,26]—the solutions

(4) $$V_{D}=\frac{V_{0}\varepsilon^{2}+\mu^{2}xt}{\varepsilon^{2}+\mu^{2}t^{2}}$$

(5) $$\rho =\frac{1}{\sqrt{\pi }{\left(\varepsilon^{2}+\mu^{2}t^{2}\right)}^{\frac{1}{2}}}exp\left[-\frac{{\left(x-V_{0}t\right)}^{2}}{\varepsilon^{2}+\mu^{2}t^{2}}\right]$$

with

(6) $$\mu =\frac{2\lambda {\left(dt\right)}^{\left[\frac{2}{f\left(\alpha \right)}\right]-1}}{\varepsilon }$$

In Equations (4)–(6), *ε* is a constant value in the definition of a parameter for the initial state densities at *t* = 0 (see below), and *V*_0_ is the initial velocity of the polymer–medicament binary structure:

(7) $$\rho \left(x,t=0\right)=\rho_{0}exp\left[-{\left(\frac{x}{\varepsilon }\right)}^{2}\right],\;\;\rho_{0}=const.$$

in the normalized coordinates system

(8) $$\xi =\frac{x}{\varepsilon },\;\;\eta =\frac{V_{0}t}{\varepsilon },\;\;\;V=\frac{V_{D}}{V_{0}},\;\;\;\phi =\frac{\rho }{\rho_{0}},\;\;\;\;\rho_{0}=\frac{1}{\pi^{\frac{1}{2}}\varepsilon }\;\;$$

For the normalized parameter

(9) $$\sigma =\frac{2\lambda {\left(dt\right)}^{\left[\frac{2}{f\left(\alpha \right)}\right]-1}}{\varepsilon V_{0}},$$

and the normalized solutions of the multifractal differential equations system [26,27] are the following:

(10) $$V\left(\xi ,\eta ,\sigma \right)=\frac{1+\sigma^{2}\xi \eta }{1+\sigma^{2}\eta^{2}}$$

(11) $$\phi \left(\xi ,\eta ,\;\sigma \right)=\frac{1}{{\left(1+\sigma^{2}\eta^{2}\right)}^{\frac{1}{2}}}exp\left[-\frac{{\left(\xi -\eta \right)}^{2}}{1+\sigma^{2}\eta^{2}}\right]$$

**Figure 1:**
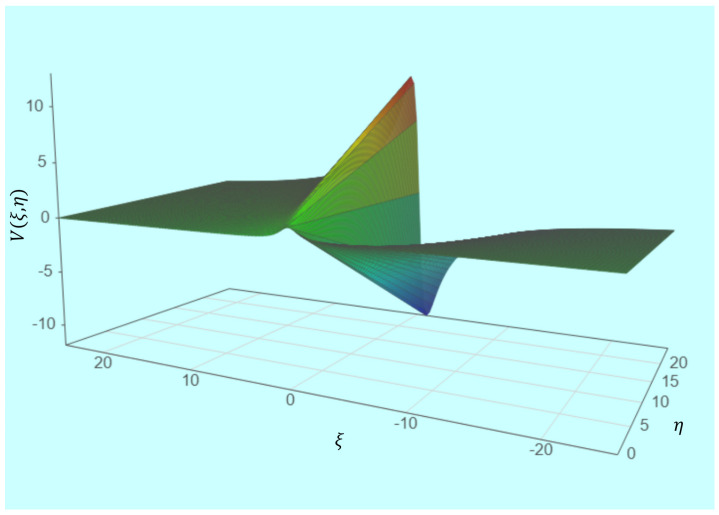
Three-dimensional graphical representations of 𝑉 (*ξ*, *η*) velocity multifractal theoretical function.

In Figure 1, the three-dimensional graphical representation of the 𝑉 (𝜉, 𝜂) velocity multifractal theoretical function in normalized coordinates for the *σ*^2^ = 1 fixed value (where the calibration is indicated) is shown.

**Figure 2:**
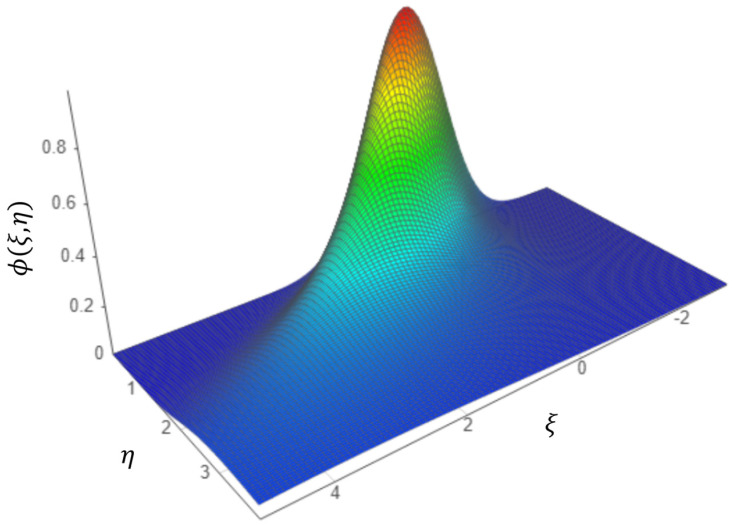
Three-dimensional graphical representation of the *ϕ* = *ϕ* (*ξ*, *η*) mass state density theoretical function.

In Figure 2, the three-dimensional graphical representation of the *ϕ* = *ϕ* (*ξ*, *η*) state density theoretical function in normalized coordinates for the *σ*^2^ = 1 fixed value (where the calibration is indicated) is shown. 

If the initial normalized mass of the polymer–medicament binary system’s constitutional unit is *μ*_0_, this permits the normalized delivered medicament mass definition as having the expression

(12) $$P\left(\eta ,\sigma ,\mu \right)=\frac{M\left(\eta \right)}{M\left(\infty \right)}=-\mu_{0}\frac{\partial \phi }{\partial \eta }$$

where *M*(*η*) coincides with the medicament mass delivered at the normalized time *η* and *M*(*∞*) is the medicament mass delivered when the time tends towards infinity, or a finite, constant quantity.

In the frequent situation in which the polymer–medicament binary system moves at a constant speed *V* ≡ 1 (for *V_D_* = *V*_0_) and *ξ* = *η*, we have the case in which Equation (12) (because of the fact that μ_0_ ≡ 1 has a fixed value) becomes

(13) $$\frac{M\left(\eta ,\sigma \right)}{M\left(\infty \right)}=\frac{\sigma^{2}\eta }{{\left(1+\sigma^{2}\eta^{2}\right)}^{\frac{3}{2}}}$$

**Figure 3:**
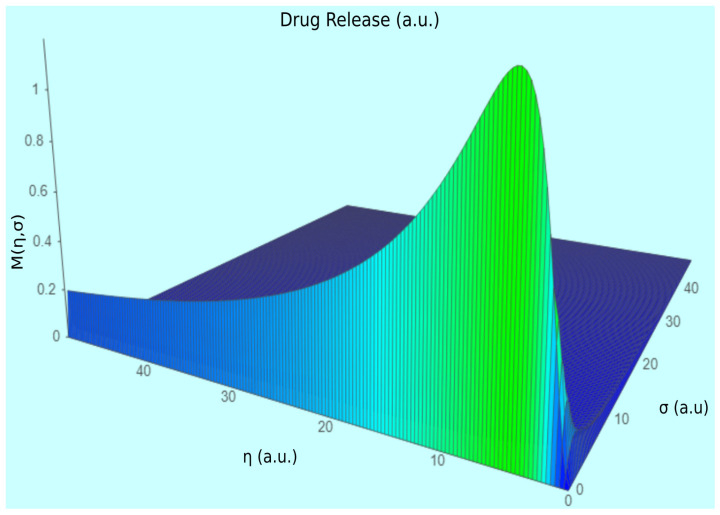
Three-dimensional plot of mass delivery theoretical function *M* (*η*, *σ*).

In Figure 3, a 3D graphical representation of drug release with a theoretical quantity/measure *M* = *M* (*η*, *σ*) into the polymer–medicament binary system, associated with normalized time *η* for fractalization at different degrees *σ*, is presented. The unit (a.u.) is the abbreviation for the arbitrary unit.

However, the idea that emerges from the study of multifractal differential equations leads to the reality derived from the “good modeling practice” of delivery kinetics, namely, that everything involves identifying the most natural boundary and initial conditions involved in the physicochemical phenomena.

**Figure 4:**
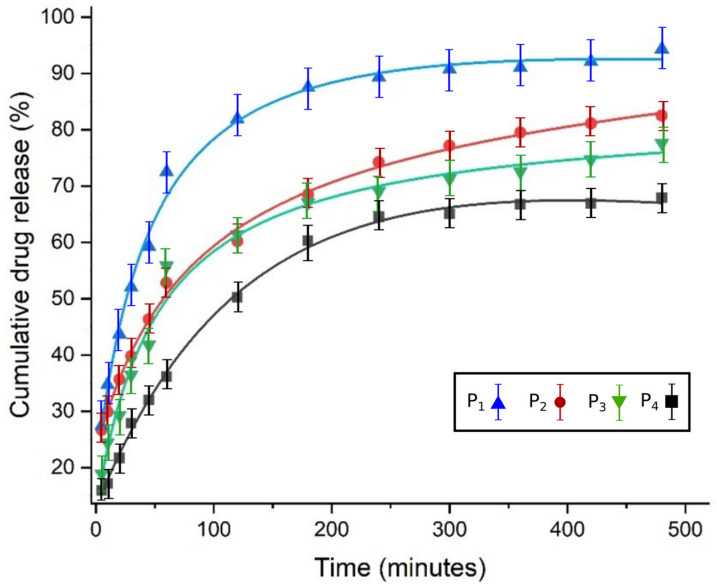
Cumulative % of drug release versus time.

In Figure 4, the silhouettes of cumulative medicament delivery (%) in vitro are presented for four distinct formulations.

The data shown in the graphic representation of Figure 4 were obtained in controlled 5-FU-chitosan binary system release experiments. As an obvious observation, it can be appreciated that the curves for cumulative drug release have different allures: one is a saturation curve (P_1_), the other two (P_2_ and P_3_) tend towards infinity for large time values, while curve P_4_ seems to decrease for long periods of time. The solid curves are the theoretical ones, according to the model used, and the experimental points are close to them, denoting a good agreement between them.

### 2.2. Box-Counting Method

The box-counting process is a reunion/assembly data procedure for analyzing repetitive complicated models and involves dividing a dataset, geometrical object, and picture into small and then smaller fragments, commonly known as “box”-form, and then analyzing the results on a scale that becomes smaller and smaller. The question now arises as to how we calculate the fractal dimension with this method [28].

Utilizing the box-counting procedure, the fractal dimension is shown by the linear regression slope where we graphically represent the *log*(*N*) value per *Y*-axis versus the log(1/*r*) value per *X*-axis, or more precisely

(14) $$d=\underset{r\to 0}{\lim}\frac{\log N\left(r\right)}{\log\left(\frac{1}{r}\right)}=-\underset{r\to 0}{\lim}\frac{\log N\left(r\right)}{\log r}$$

An identical formula is utilized to determine the fractal dimension for computational applications of the fractal dimension to any strictly self-similar fractals. Stricto sensu, the size in question is the extent of the total fractals enclosed/inserted in a standard Euclidean space. 

### 2.3. Lacunarity 

Lacunarity makes a natural couple with the fractal dimension and is best used to depict the surface quality of a fractal object, including cracks (holes) and everything else. More specifically, it refers to the homogeneities and inhomogeneities of the texture in a global vision, with the hole statistics and their size as the distribution function. In fractal analysis, the lacunarity interprets the measure of present gaps (porous texture) or “real texture” measure [29].

It is thus ascertained as the inhomogeneity degree and translational (2D) and rotational (3D) invariance of the surface picture, wherein reduced/small lacunarity assumes homogenous existence and the rotating image changes the insignificant context. Thus, lacunarity is a concept different and separate from the fractal dimension. It has no connection with fractal topology, and more numerical variables are needed for complete decisions. This fractal measure is loudly connected with the gap size distribution of the fractal object and with its deviation from standard translational invariance. Generally, a fractal is most lacunar if its gaps are disposed to be great, as they comprise wide surface zones.

(15) $$\Lambda \left(\varepsilon \right)=\frac{Z^{\left(2\right)}}{{\left(Z^{\left(1\right)}\right)}^{2}}$$

(16) $$Z^{\left(1\right)}=\underset{\varepsilon }{\sum }PQ\left(P,\varepsilon \right)$$

(17) $$Z^{\left(2\right)}=\underset{\varepsilon }{\sum }P^{2}Q\left(P,\varepsilon \right)$$

(18) $$Q\left(P,\epsilon \right)=\frac{n\left(P,\varepsilon \right)}{N\left(M,\epsilon \right)}$$

In the formulas above: size of the map = *M*; size of the box = *ε*; the box mass = *P*; *n* (*P*, *ε*) is the number of boxes containing P object pixels; N (M, ε) = (M-ε+1)^2^ is the number of possible box positions; and *Q*(*P*, *ε*) is the probability calculated by Equation (1). At the same time, *P*•*Q*(*P*, *ε*) and *P*^2^•*Q*(*P*, *ε*) are the first and second moments, while Z^(1^) and Z^(2)^ are the sum of the first and second moments, calculated by Equations (3) and (4), respectively. Equation (2) is the lacunarity $\Lambda \left(\varepsilon \right)$ of the dataset for box size *ε*.

### 2.4. Assessment of Scanning Electron Microscope Images Using Fractal Analysis 

The obtained formulations were noted with P_1_, P_2_, P_3_, and P_4_. The number associated with each letter/compound is appropriate to the molar proportion of the amino/aldehyde class, i.e., 1:1, 2:1, 3:1, and 4:1, respectively. 

The 5-fluorouracil in our formulations was determined by polarized light microscopy (Figure 1), which disclosed the medicament’s evident segregation in the hydrogels with large, reticulated density (P_1_, P_2_), while for the hydrogel compounds with lower reticulated density (P_4_), a birefringent, granular structure was observed, characteristic of crystal submicrometric dimensions distributed below the apparatus detection tolerance [30]. 

**Figure 5:**
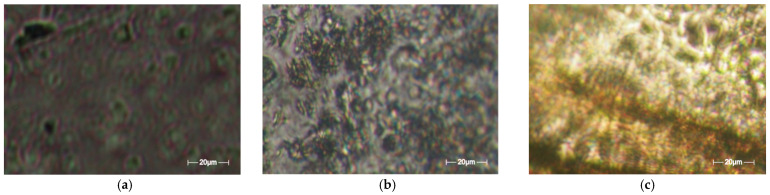
Typical POM pictures of the compounds/formulations: (**a**) POM-P_1_; (**b**) POM-P_2_; (**c**) POM-P_4_.

In Figure 5, representative POM typical images are shown. More precisely, there are three POM images of the three samples obtained, each with a different concentration: P_1_, P_2,_ and P_4_. The scale bar for the POM photographic images is 20 microns. Figure 5a shows POM-P_1_, Figure 5b shows POM-P_2_, and Figure 5c shows POM-P_4_.

**Figure 6:**
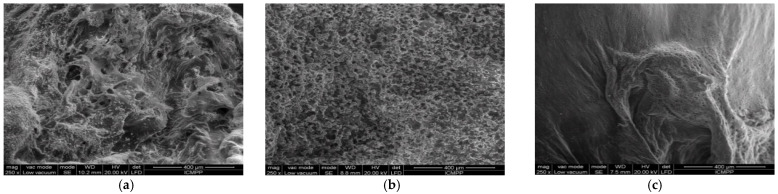
Typical SEM pictures of the compounds/formulations: (**a**) SEM-P_1_; (**b**) SEM-P_2_; (**c**) SEM-P_3_.

In Figure 6, representative SEM images are shown. More precisely, there are three SEM images of the three obtained samples, each with a different concentration (P_1_, P_2,_ and P_3_). The scale bar for the SEM photographic images is 400 microns. Figure 6a shows SEM-P_1_, Figure 6b shows SEM-P_2_, and Figure 6c shows SEM-P_3_.

The morphology of these three obtained formulations—P_1_, P_2,_ and P_3_—was investigated by scanning electron microscopy, and was then evaluated. Strictly speaking, in this paper, a new way of interpreting the SEM images of the samples is presented (fractal analysis), which is the main novelty of this paper compared to [8].

The fractal dimensions and gap orientations/distributions on the studied surfaces (lacunarity) of the studied SEM pictures were computed via the well-known method of fractal analysis [31]. In an attempt to determine a fine punctual context, i.e., a correct inventory at the pixel level, recent computational software for the investigation of complex neural diseases using CT and MRI pictures were utilized [32,33,34].

As can be observed in the SEM images, the formulations have a distinct porous structure, as can be seen by the presence of evident medicament crystals enclosed in the orifices/pores of the walls (Figure 6). The medicament crystal caliber decreased as the crosslinking level decreased, conforming with SEM image monitoring, as mentioned in relation to chitosan-founded formulations in the literature [32]. For the quality valuation of the surface captured on the SEM images, fractal geometry indicators were applied, which led to the calculation of the fractal dimension (a ratio providing a statistical index of complexity) of the respective image, as well as it’s lacunarity [33]. The voxel graphical representations for each SEM picture are also referenced individually in this paper. 

**Figure 7:**
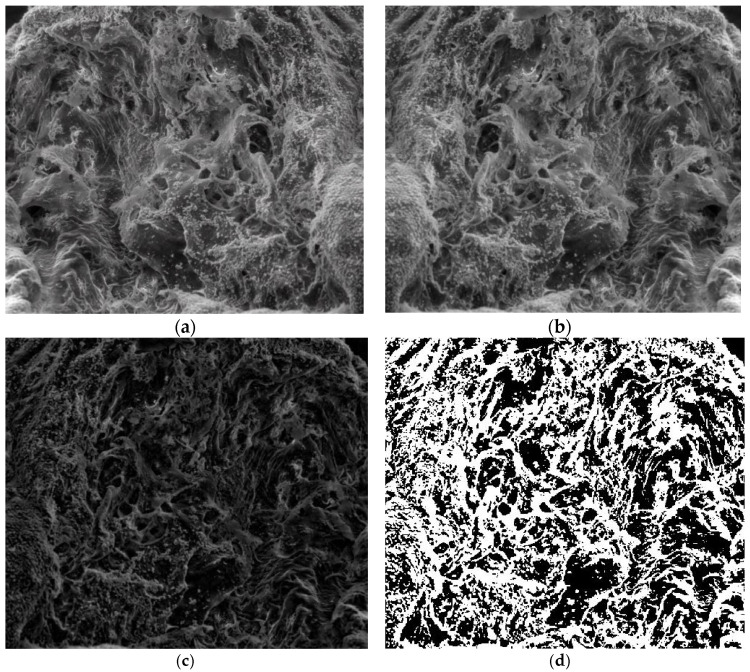
Processing stages of P_1_ image. (**a**) Original image (the entire portion); (**b**) grayscale version; (**c**) grayscale version without luminance; (**d**) binarized version.

Figure 7a is the original image P_1_ of the entire portion, Figure 7b is the grayscale version of the original image, Figure 7c is the grayscale version of the image without luminance, and Figure 7d is the binarized version image without luminance.

Figure 7 shows the three phases of processing the original P_1_ image in order to apply the fractal analysis procedure and calculate the fractal dimension and lacunarity. For image binarization, a threshold of 30 units was utilized.

Via the numerical assessment of the chosen picture (P_1_) with fractal analysis software [35], it was found that the fractal dimension value D = 1.8621 had a standard deviation of $s=\pm \sqrt{\sigma^{2}}=\pm 0.0733$ and a lacunarity value of Λ = 0.0385, as shown in Table 1.

**Figure 8:**
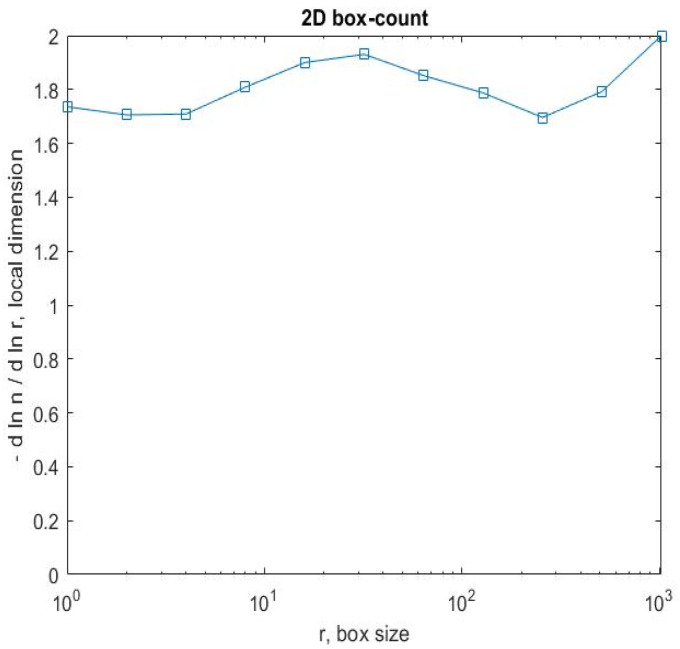
Two-dimensional box-count algorithm: fractal dimension.

**Figure 9:**
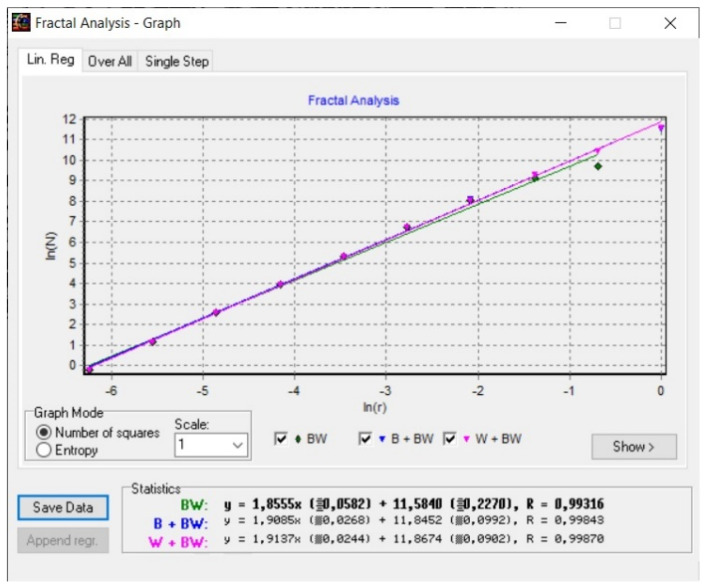
Graphic of fractal dimension for elected P_1_ picture zone.

Figure 8 shows a 2D graphic of the fractal dimension using the box-counting method. It can be seen that the fractal dimension is between 1.73 and 2. Figure 9 presents an inspection of the elected P_1_ picture zone (fractal dimension computation) with the Harmonic and Fractal Image Analyzer Demo computer program (Prague, Czech Republic), version 5.5.30 [36]. The fractal dimensions of the different ruler scales are equal to *r*.

**Figure 10:**
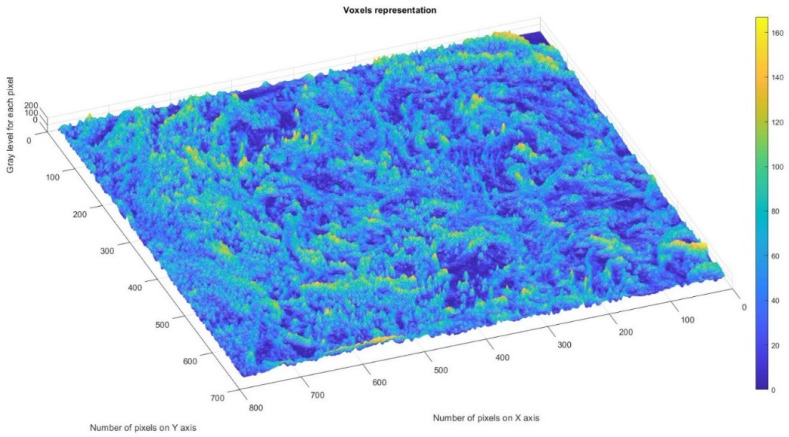
Three-dimensional voxel representation of P_1_ image.

Figure 10 shows the voxels of the analyzed P_1_ image and a 3D graphic representation. The gray level is shown on the *oZ* axis, while the corresponding numbers of pixels are on the other two axes (*oX* and *oY*) [37]. 

In a given three-dimensional (3D) graphical representation, a voxel expresses a value in a normal network in a 3D space. In the case of pixels in a two-dimensional bitmap, locations (coordinates) are not usually coded/fixed by the values represented as belonging to each of them. Conversely, associated interpretation systems deduct the voxel’s real position in relation to its relative position, compared to the other voxels. Geometrical position in terms of data organization thus results in a unique volumetric image.

**Figure 11:**
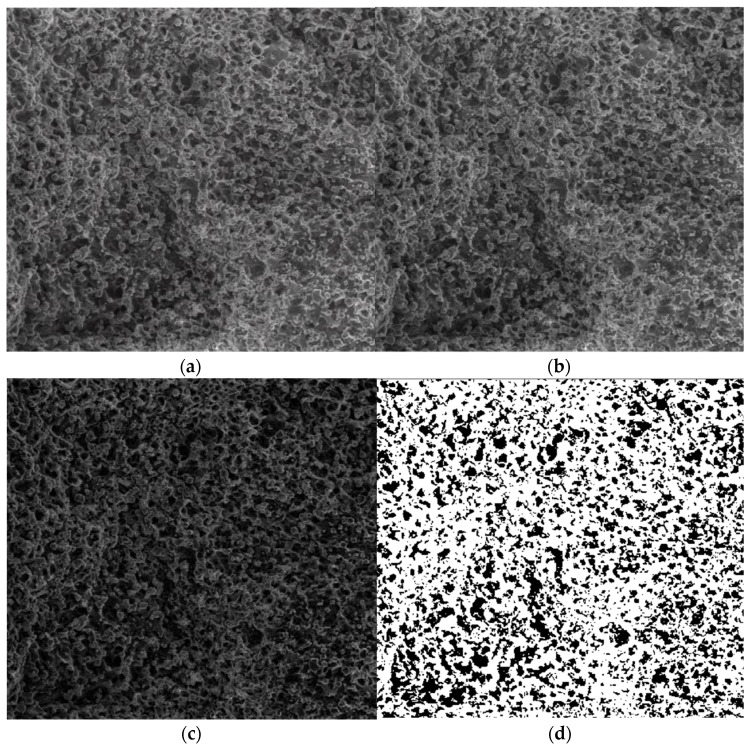
Processing stages of the P_2_ image. (**a**) Original image (the entire portion); (**b**) grayscale version; (**c**) grayscale version without luminance; (**d**) binarized version.

In Figure 11, the operation stages of the P_2_ SEM image are presented. In Figure 11a, the original image of the entire portion is depicted, the grayscale version of the original image is shown in Figure 11b, the grayscale version of the image without luminance is shown in Figure 11c, and Figure 11d is the binarized version of the image without luminance. 

Figure 11 shows the three phases of processing the original P_2_ image in order to apply the fractal analysis procedure and calculate the fractal dimension and lacunarity. For image binarization, a threshold of 25 units was utilized.

Via the numerical assessment of the chosen picture (P_2_) with fractal analysis software [35], it was found that the fractal dimension value *D* = 1.8837 had a standard deviation of $s=\pm \sqrt{\sigma^{2}}=\pm 0.0894$ and a lacunarity value of Λ=0.0498, as shown in Table 2.

**Figure 12:**
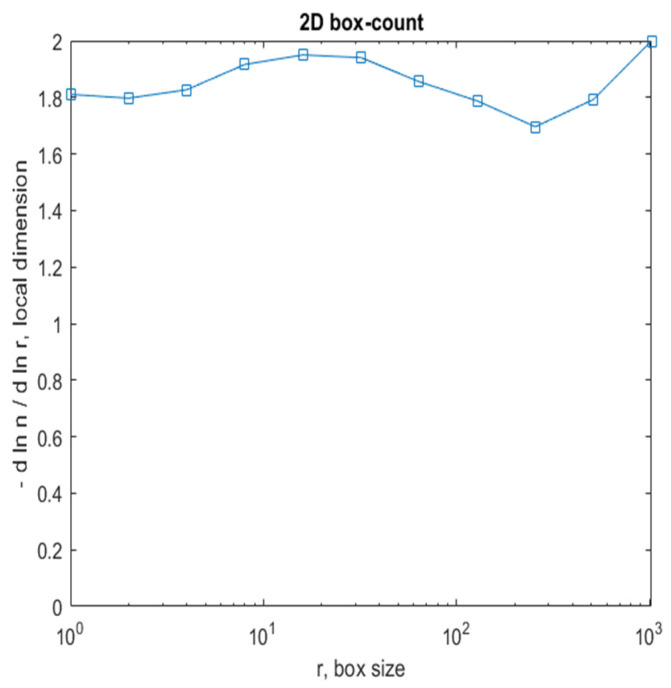
Two-dimensional box-count algorithm: fractal dimension.

**Figure 13:**
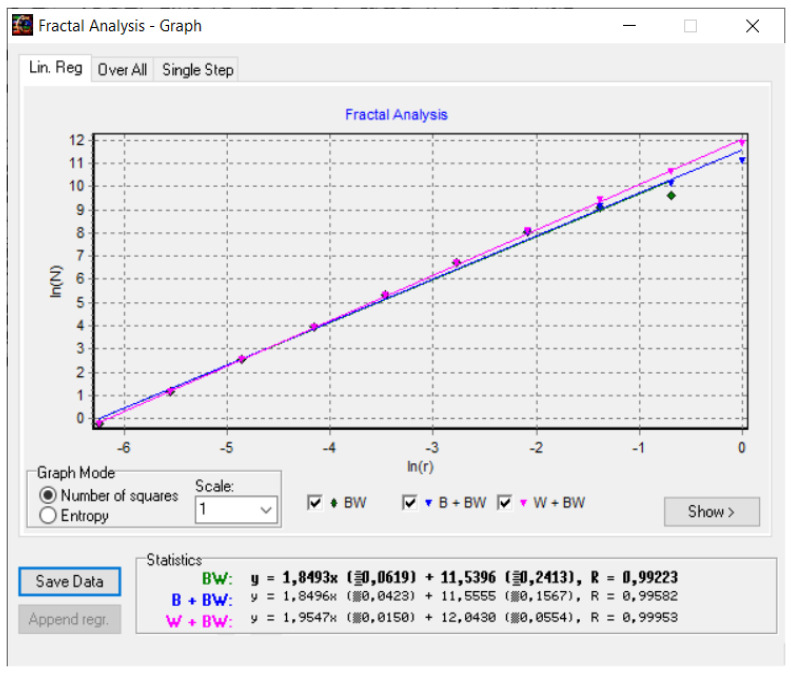
Graphic of fractal dimension for elected P_2_ picture zone.

Figure 12 shows a 2D graphic of the fractal dimension using the box-counting method. It can be seen that the fractal dimension is between 1.8 and 2. Figure 13 presents an inspection of the elected P_2_ picture zone (fractal dimension computation) with the Harmonic and Fractal Image Analyzer Demo program, version 5.5.30 [36]. The fractal dimensions of the different ruler scales are equal to *r*.

**Figure 14:**
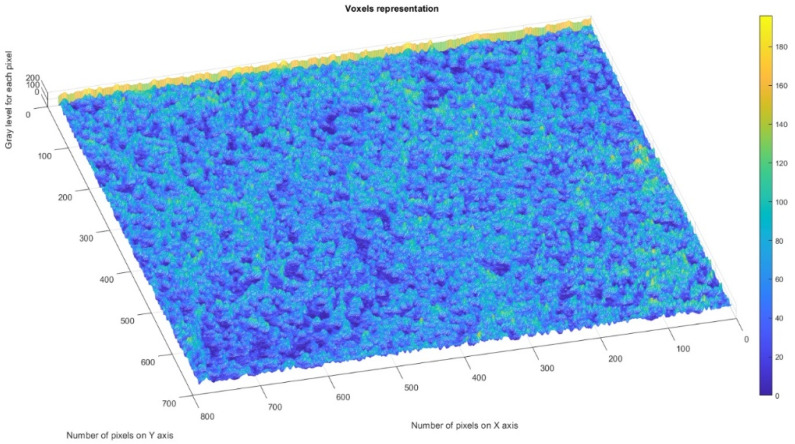
Three-dimensional voxel representation of P_2_ image.

Figure 14 shows the voxels of the analyzed P_2_ image and a 3D graphical representation. The gray level is shown on the *oZ* axis, while the corresponding numbers of pixels are on the other two axes (*oX* and *oY*) [37]. 

**Figure 15:**
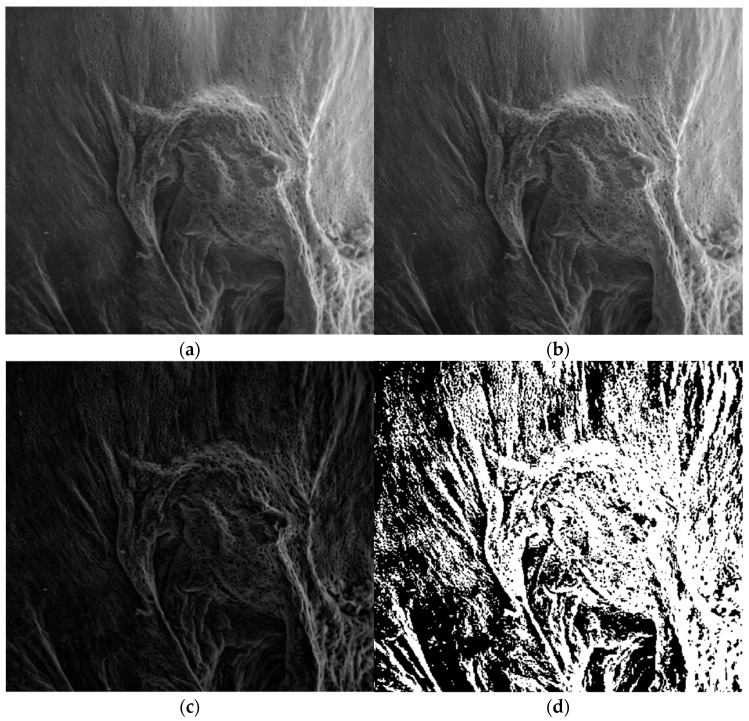
Processing stages of the image P_3_. (**a**) Original image (the entire portion); (**b**) grayscale version; (**c**) grayscale version without luminance; (**d**) binarized version.

In Figure 15, the operation stages of the P_3_ SEM image are presented. In Figure 15a, the original image of the entire portion is shown, the grayscale version of the original image is shown in Figure 15b, the grayscale version of the image without luminance is shown in Figure 15c, and Figure 15d is the binarized version of the image without luminance. 

In Figure 15, the three phases of processing the original P_3_ image are shown in order to apply the fractal analysis procedure and calculate the fractal dimension and lacunarity. For image binarization, a threshold of 10 units was utilized.

Via the numerical assessment of the chosen picture (P_3_) with fractal analysis software [35], it was found that the fractal dimension value *D* = 1.8561 had a standard deviation of $s=\pm \sqrt{\sigma^{2}}=\pm 0.0702$ and a lacunarity value of Λ=0.0324, as shown in Table 3.

**Figure 16:**
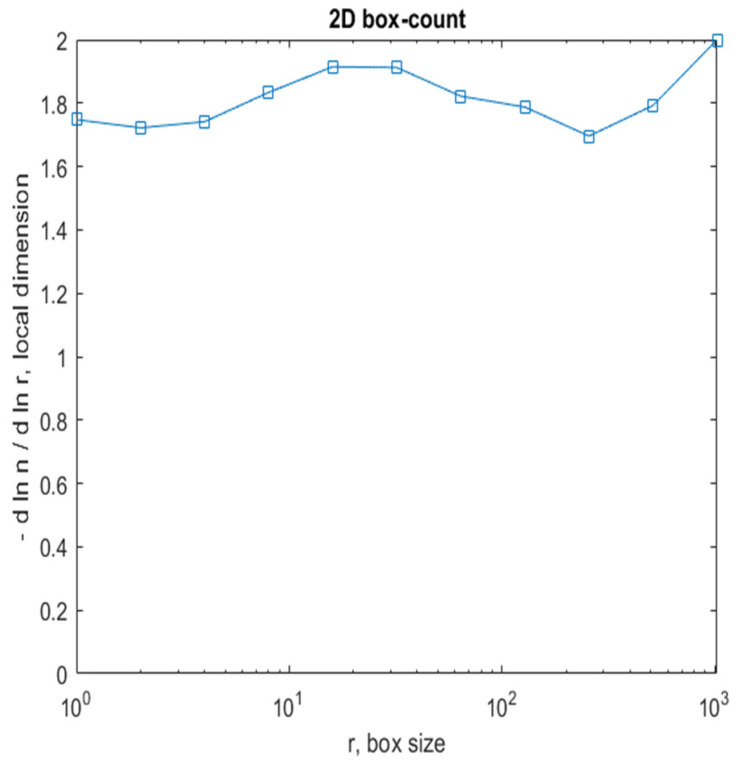
Two-dimensional box-count algorithm: fractal dimension.

**Figure 17:**
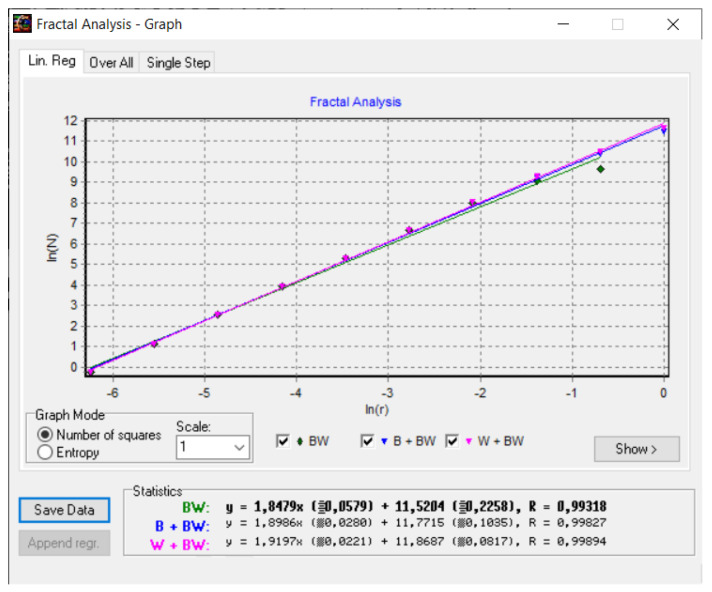
Graphic of fractal dimension for elected P_3_ picture zone.

Figure 16 shows a 2D graphic of the fractal dimension using the box-counting algorithm method. It can be seen that the fractal dimension is between 1.75 and 2. Figure 17 presents the inspection of the elected P_3_ picture zone (fractal dimension computation) with the Harmonic and Fractal Image Analyzer Demo program, version 5.5.30 [36]. The fractal dimensions of the different ruler scales are equal to *r*.

**Figure 18:**
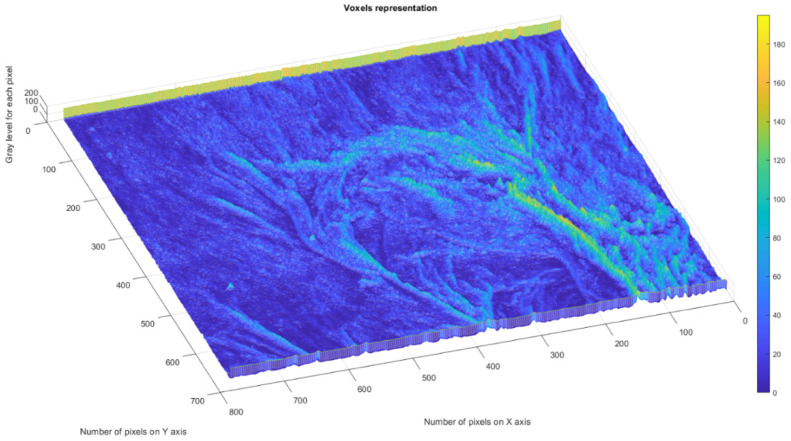
Three-dimensional voxel representation of P_3_ image.

Figure 18 shows the voxels of the analyzed P_3_ image and a 3D graphical representation. The gray level is shown on the *oZ* axis, while the corresponding numbers of pixels are on the other two axes (*oX* and *oY*) [37]. 

In Figure 8, Figure 12 and Figure 16, graphical representations for determining the fractal dimensions depending on the box size *r* (via the box-counting method) are introduced.

Figure 9, Figure 13 and Figure 17 show the 2D graphical depictions of the linear regression slope findings for fractal dimension computation. Figure 10, Figure 14 and Figure 18 show the voxel 3D representation graphs for the pictures P_1_, P_2_, and P_3_ from the amended zone. The three coordinate axes are assigned as follows: the pixel number is on the ox axis, the pixel number is on the oy axis, and the gray intensity level for the respective pixel is on the oz axis. In line with the generated computer graphics, the so-called voxel shows the numerical amount/value directly connected to the regular grid in a 3D spatial configuration.

## 3. Conclusions

In this paper, 5-fluorouracil-encapsulated chitosan nanoparticles were realized for the investigation of the release of spatially delimited medicaments, considering the pH of chitosan nanoparticles’ sensibility. 

To correctly analyze the dynamic behavior of 5-fluorouracil delivery with chitosan nanoparticles, in vitro delivery information was investigated utilizing a multifractal kinetic equation. The developed mathematical multifractal model has been confirmed by the experimental measurements corresponding to 5-fluorouracil release outside the chitosan-formed matrix. 

The increase in the efficiency of chitosan and 5-fluorouracil-encapsulated elements resulted in nanometer values of particle dimensions and measurement distributions. This was certified through scanning electron microscopy (SEM) observations. The P_1_, P_2,_ and P_3_ SEM images of the three formulations were found to conform with the fractal analysis procedures, and the fractal dimension and lacunarity values were calculated. Thereby, the P_1_ image had the fractal dimension value of D = 1.8621 ± 0.0733 and the lacunarity value of Λ = 0.0385. The P_2_ image had the fractal dimension value of D = 1.8837 ± 0.0894 and the lacunarity value of Λ = 0.0498. Finally, image P_3_ had the fractal dimension value of D = 1.8561 ± 0.0702 and the lacunarity value of Λ = 0.0324.

## 4. Materials and Methods

### 4.1. Materials

Chitosan-covered magnetic nanoparticles were utilized to expand the deliverance of 5-fluorouracil [4]. Chitosan (243 kDa, DA: 87%), 3,7-dimethyl-2,6-octadienal (95%), 5-fluorouracil (purity: 99%), and phosphate tampon solution with a pH of 7.4 were purchased from Aldrich Chemical Co Inc. and brought to the required standard quality. All other reagents were of analytical grade.

### 4.2. Formulation Preparation

All the formulations (i.e., experimental products) obtained were adapted and realized through chitosan hydrogelation (in situ), starting with 3,7-dimethyl-2,6-octadienal and 5-fluorouracil as a direct consequence of a known protocol [21]. In a short time, 3,7-dimethyl-2,6-octadienal (2% solution) was blended into 5-fluorouracil and was dripped into the chitosan (3% solution) in an aqueous solution (1%) of dissolved acetic acid. The 5-fluorouracil element, enclosed in the chitosan nanoparticle capsules, was used to examine the localized medicament release potential of the pH sensibility of the utilized chitosan nanoparticles. The maximum reported levels of chitosan and 5-fluorouracil in the encapsulated particles were 150 nm and 250 nm in terms of particle dimension (so nanoparticles). The measurement distributions centered on the particles’ small size, as confirmed via the scanning electron microscopy investigations. The provocation launched on this occasion will be examined by the medicament release of 5-fluorouracil-encapsulated chitosan particles with various pH modifications. According to the observations made, the deliverance of 5-fluorouracil from various hydrogel compounds resulted in zero-order kinetics. These obvious consequences suggest that chitosan hydrogel performs a significant function in controlling medicament deliverance to circumambient tissues.

As an immediate observation, the hydrogelation period augmented as the aldehyde quantity reduced. This happened immediately for the 1/1 molar proportion of the amine/aldehyde functional class and slowly continued for a 24-h period in the case of the 4/1 molar proportion. 

The principal purpose was to realize chitosan polymeric products via a solvent evaporation emulsification procedure by utilizing various polymer proportions. Ultimately, the achieved hydrogels were lyophilized and then analyzed from a physico-chemical point of view.

### 4.3. Methods

The total gelation period was established when it was visually observed that the formed chemical blend was converted from a viscous consistency to a rubbery consistency. The xerogels were realized by lyophilization from the equivalent hydrogels, utilizing a Labconco FreeZone Freeze Dry device (FreeZoner2.5 Liter Freeze Dry laboratory apparatus) for one day (24 h) in temperature conditions of −50 °C and a pressure of 0.04 mbar.

Optimized polymer proportions were determined by appropriate experimental methods, including differential scanning calorimetry (DSC), X-ray diffraction (XRD), entrapment capability and particle dimension, and likeliness to succeed in enteric covering. Polarized optical microscopy pictures were achieved with a Zeiss Axio Imager M2 microscope, and hydrogels and xerogels were the compounds used in this experiment. The hydrogel morphology modifications were observed with an SEM EDAX -Quanta 200 field emission scanning electron microscope, and manipulated at an electric tension of 20 keV.

## Figures and Tables

**Table 1:** Fractal characteristic calculation of picture P_1_.

Name	Fractal Dimension	Standard Deviation	Lacunarity
Image P_1_	1.8621	0.0733	0.0385

**Table 2:** Calculation of fractal characteristics of P_2_ picture.

Name	Fractal Dimension	Standard Deviation	Lacunarity
Image P_2_	1.8837	0.0894	0.0498

**Table 3:** Calculation of fractal characteristics of picture P_3_.

Name	Fractal Dimension	Standard Deviation	Lacunarity
Image P_3_	1.8561	0.0702	0.0324

## Data Availability

The data used to support the findings of this study cannot be accessed due to commercial confidentiality.
